# Adaptation of *Lacticaseibacillus rhamnosus* CM MSU 529 to Aerobic Growth: A Proteomic Approach

**DOI:** 10.3390/microorganisms11020313

**Published:** 2023-01-25

**Authors:** Tatiana Yu. Dinarieva, Alena I. Klimko, Jörg Kahnt, Tatiana A. Cherdyntseva, Alexander I. Netrusov

**Affiliations:** 1 Faculty of Biology, Moscow State University, 119234 Moscow, Russia; 2 Core Facility for Mass Spectrometry & Proteomics, Max Planck Institute for Terrestrial Microbiology, Karl-von-Frisch-Straße 10, 35043 Marburg, Germany; 3 Faculty of Biology and Biotechnology, Higher School of Economics, 101000 Moscow, Russia

**Keywords:** *Lacticaseibacillus rhamnosus*, aerobic conditions, oxidative stress, pyruvate oxidase, pyridoxine 5’-phosphate oxidase, NADH peroxidase, alkyl hydroperoxide reductase C, acetolactate synthase

## Abstract

The study describes the effect of aerobic conditions on the proteome of homofermentative lactic acid bacterium *Lacticaseibacillus rhamnosus* CM MSU 529 grown in a batch culture. Aeration caused the induction of the biosynthesis of 43 proteins, while 14 proteins were downregulated as detected by label-free LC-MS/MS. Upregulated proteins are involved in oxygen consumption (Pox, LctO, pyridoxine 5’-phosphate oxidase), xylulose 5-phosphate conversion (Xfp), pyruvate metabolism (PdhD, AlsS, AlsD), reactive oxygen species (ROS) elimination (Tpx, TrxA, Npr), general stress response (GroES, PfpI, universal stress protein, YqiG), antioxidant production (CysK, DkgA), pyrimidine metabolism (CarA, CarB, PyrE, PyrC, PyrB, PyrR), oligopeptide transport and metabolism (OppA, PepO), and maturation and stability of ribosomal subunits (RbfA, VicX). Downregulated proteins participate in ROS defense (AhpC), citrate and pyruvate consumption (CitE, PflB), oxaloacetate production (AvtA), arginine synthesis (ArgG), amino acid transport (GlnQ), and deoxynucleoside biosynthesis (RtpR). The data obtained shed light on mechanisms providing O_2_-tolerance and adaptation to aerobic conditions in strain CM MSU 529. The biosynthesis of 39 from 57 differentially abundant proteins was shown to be O_2_-sensitive in lactic acid bacteria for the first time. To our knowledge this is the first study on the impact of aerobic cultivation on the proteome of *L. rhamnosus*.

## 1. Introduction

Lactic acid bacteria (LAB) are widely used as starter and probiotic cultures in the food industry. Probiotics as functional food ingredients benefit human and animal health. Probiotics have great potential in clinical applications as well, treating allergies, lactose intolerance, dental caries, hypercholesterolemia, gastroenterititis, and inflammatory bowel disease [1]. Among different species of lactobacilli considered as probiotics, *Lacticaseibacillus rhamnosus* is intensively studied. *L. rhamnosus* strains are able to survive in a gastrointestinal tract due to adhesion to the epithelial cells of intestines followed by biofilm formation, which subsequently competitively suppresses the attachment and growth of pathogenic microorganisms [2]. *L. rhamnosus* J10-L and *L. rhamnosus* GG (LGG) revealed a strong antibacterial activity towards *Shigella sonnei* in vitro due to the production of the lactic acid [3]. *L. rhamnosus* strains prevented the gastrointestinal tract colonization by *Candida* species [4,5]. *L. rhamnosus* L34 suppressed the production of interleukin-8, a mediator of an inflammatory response, by colonic epithelial cells induced by *Clostridium difficile* causing hospital-acquired diarrhea and colitis [6]. LGG could modulate M1 macrophage polarization in vitro to prevent *Salmonella Typhimurium* infection, supporting its role in immune regulatory activity [7]. LGG, generally recognized as safe by the U.S. Food and Drug Administration with qualified presumption of safety (European Food Safety Authority), is widely used in the food industry of fermented dairy products and probiotic production all over the word [8]. The search of new safe strains with probiotic properties and high stability to conditions of technological processes is of current interest today.

Aerobic and respiratory cultivations are considered to be the technologically advanced ways to obtain starter and probiotic cultures of LAB. In the case of respiratory cultivation, LAB strains are grown aerobically in the presence of exogenous hemin (lactococci) or hemin plus menaquinone (lactobacilli). Aerobic/respiratory cultivation has some advantages for the industrial process and biotechnological applications compared to anaerobic cultivation. Aerobically/respiratory grown cells demonstrate enhanced stability to oxidative stress, freezing, freeze-drying, and prolonged storage [9,10,11]. They differ by higher production of aroma compounds, and in the case of respiratory grown cultures, by increased biomass yields. LAB belong to oxygen-tolerant anaerobes with fermentative metabolism. They lack genes of the complete heme biosynthesis pathway; only a few representatives of *Lactococcus*, *Enterococcus*, *Leuconostoc*, and *Weissella* are able to synthesize quinone [12]. Some LAB possess an active electron transport chain (ETC) consisting of NADH (reduced nicotinamide adenine dinucleotide) dehydrogenase, menaquinone, and quinol oxidase *bd* when grown under respiratory conditions. Aerobic and respiratory metabolism has been intensively studied in *Lactococcus lactis*, *Lactiplantibacillus plantarum*, and *Lacticaseibacillus casei* [9,10,11,12,13]. The impact of aerobic and respiratory conditions on LAB life-style was elucidated at the transcriptomic level in *Lc. lactis* [14,15], *Lp. plantarum* [16], and *Lentilactobacillus buchneri* CD034 [17], as well as at the proteomic level in *Lc. lactis* [18], *Lp. plantarum* WCFS1 [19], *L. casei* N87 [20], *Carnobacterium* and *Leuconostoc* species [21].

In this study, we describe the effect of aerobic conditions in a batch culture on the proteome of *Lacticaseibacillus rhamnosus* CM MSU 529, a member of the *L. casei* group. Recently, we have demonstrated the functioning of ETC consisting of NADH dehydrogenase 2, menaquinone, and quinol oxidase *bd* in *L. rhamnosus* CM MSU 529 [22]. NADH oxidase activity of membrane preparations, obtained from cells grown aerobically with hemin supplementation, increased by 4.6-fold in the presence of exogenous vitamin K_2_. This study reveals the mechanisms of oxygen tolerance and adaptation to the aerobic growth of strain CM MSU 529. Protein abundance patterns imposed by aerobic growth are strain-unique in LAB. The expression of 39 from 57 quantitatively altered proteins in strain CM MSU 529 was shown to be O_2_-sensitive in LAB for the first time. To our knowledge this is the first study on the effect of aerobic cultivation on the proteomic profile of *L. rhamnosus*.

## 2. Materials and Methods

### 2.1. Cultivation of L. rhamnosus CM MSU 529

*L. rhamnosus* CM MSU 529 used in the study was isolated from a feces sample of a 4-month-old infant at the Microbiology Department of Moscow State University. Strain CM MSU 529 was grown in an MRS medium (Merck KGaA, Darmstadt, Germany), pH 6.5, in a batch culture under static and aerobic conditions. Static cultures were grown in 12 mL vials closed by rubber stoppers and aluminum caps with 10 mL of the medium at 37 °C for 24 h. Aerobic cultures were grown in 250 mL flasks with 15 mL of the medium at 37 °C and 200 rpm for 24 h; 24 h statically grown cultures were used as inoculum (10% *v*/*v*). Cells were harvested at the late exponential phase of growth by centrifugation (4000 rpm, 10 min, 4 °C) and washed 3 times with deionized water. The obtained pellets were washed 3 times (4000 rpm, 10 min, 4 °C) with 1 mL of cold acetone, dried under air, and stored at −20 °C.

### 2.2. Protein Extraction

Acetone dried cells (4.0–5.6 mg) were lysed by incubation in 300 μL of a 2% (*w*/*v*) sodium deoxycholate (DOC) in 100 mM ammonium bicarbonate (ABC) solution at 90 °C for 50 min at 500 rpm followed by centrifugation (15,000 rpm, 5 min). The total protein was determined in supernatants using a Pierce bicinchoninic acid protein assay kit (Thermo Fisher Scientific GmbH, Dreieich, Germany) [23]. The cysteines were reduced by 5 mM Tris(2-caboxyethyl)phosphine and incubated for 10 min at 90 °C. The samples were briefly cooled on ice and carbamidomethylated in the presence of 10 mM iodoacetamide for 30 min at 25 °C in the dark.

### 2.3. Protein Digest

Aliquots containing 50 µg of dissolved protein were taken from each sample and diluted with 100 mM ABC to a final concentration of 0.42% DOC. The obtained samples were digested with 1 µg of MS approved trypsin (Serva Electrophoresis GmbH, Heidelberg, Germany) at 30 °C for 12 h. The DOC was then precipitated by acidification with trifluoroacetic acid (TFA) and subsequent centrifugation (15,000 rpm, 20 min, 4 °C).

### 2.4. Liquid Chromatography-Mass Spectrometry

Tryptic peptide solutions were desalted using C18-SPE (Microspin column C18 WP, 20 mg Chromabond; Macherey-Nagel GmbH & Co. KG, Düren, Germany) according to the manufacturer’s instructions. Eluates were evaporated under vacuum, suspended in 100 µL of 0.1% TFA (*v*/*v*), and analyzed by liquid chromatography-mass spectrometry (LC-MS) on an Orbitrap Exploris 480 Mass Spectrometer connected to an Ultimate 3000 rapid-separation liquid chromatography nano instrument (Thermo Fisher Scientific GmbH). Peptide separation was performed on a reverse-phase high-performance liquid chromatography column (75 μm × 40 cm) packed in-house with C18 resin (2.4 μm; Dr. Maisch, Ammerbuch, Germany) with a 60 min linear gradient from 6 to 50% of solvent B at a flow rate of 0.3 µL/min. The following solvents for separation were used: solvent A, 0.1% (*w*/*v*) formic acid; solvent B, 0.1% (*w*/*v*) formic acid/80% (*v*/*v*) acetonitrile. 

### 2.5. Data Acquisition

MS spectra were acquired at a resolution of 60,000 over a mass-to-charge range of 350–1650 m/z using a maximum injection time of 25 ms. MS scans were followed by tandem MS/MS scans. To increase the efficiency of MS/MS scans, precursor ions that were unassigned, singly charged or with charge states > 6 were excluded. The dynamic exclusion duration was set to 14 s. Peptide precursors were isolated (isolation window 1.5 m/z), fragmented using HCD collision energy of 27%, and analyzed at a resolution of 15,000.

### 2.6. Peptide Identification, Quantification, and Statistical Analysis

MS data were searched against an in-house protein database using Sequest embedded into Proteome Discoverer 1.4 software (Thermo Fisher Scientific GmbH). The resulted reference was the *L. rhamnosus* LRHMDP2 proteome (Uniprot proteome ID: UP000009330). The obtained msf-files were combined into a summary table using Scaffold (v. 5.0.1). The identification parameters for peptides were as follows: trypsin digestion with maximum of 2 missed cleavage sites; peptide length of 7–144 amino acids; maximum delta Cn − 0.05; the precursor mass tolerance and fragment mass tolerance were ±10 ppm and ±0.02 Da, respectively; spectrum matching requirements were set to 1 for mass of b and y ions; carbamidomethyl cysteine (+57.021 Da) was set as a static modification; dynamic modifications with oxidation (+15.995 Da) of the Met residues and deamidation (+0.984 Da) of the Asn and Gln residues. Protein and peptide FDR were set to 0.01 (1%) and the minimum peptide number was 1 for the identified proteins. 

Furthermore, MS data evaluation and statistical analysis were performed using MaxQuant (v. 2.0.1.0) and SaveQuant (v. 2.3.5). Proteins of score > 75, of fold change 1.5 times (log_2_ fold change ≥ 0.6 or ≤ −0.6) with *p* and *q* values ≤ 0.05 were considered to be differentially expressed in response to aeration versus static growth. Protein fold changes were estimated using mean intensities measured in samples of the three independent cultivations (*cv* ≤ 0.3).

Identified proteins were clustered according to their biological functions applying the database of Clusters of Orthologous Groups (COG) (http://www.ncbi.nlm.nih.gov/COG, accessed on 1 August 2022) [24]. Protein functions were obtained from https://w.w.w.uniprot.org web resource, accessed on 3 August 2022.

### 2.7. Cell Dry Weight Determination

The 10–15 mL cultures were harvested by centrifugation (4000 rpm, 10 min, 4 °C) and washed 3 times with deionized water. Mass of centrifuge tubes, empty or with biomass pellets, was adjusted to a constant weight at 90 °C. Cell dry weights (or pH values of culture supernatants) are presented as “mean ± standard deviation” calculated using Microsoft Excel (Microsoft, Redmond, WA, USA) on the basis of the data obtained from the three independent cultivations.

### 2.8. Protein–Protein Interaction Analysis

Protein–protein interactions were analyzed using a STRING (v. 11.5) web resource (https://string-db.org/, accessed on 29 November 2022); 53 of 57 differentially abundant proteins of strain CM MSU 529 were identified in LGG proteome to obtain a full STRING network. The following basic settings were applied: The edges indicate both functional and physical protein associations with a confidence cutoff of 0.7; active interaction sources included textmining, experiments, databases, co-expression, neighborhood, gene fusion, and co-occurrence; only associated proteins are shown. Protein–protein network was clustered using MCL inflation parameter of 1.1.

## 3. Results

### 3.1. Effect of Aeration on the Proteome of Strain CM MSU 529

The intensive aeration significantly affected the proteomic profile of *L. rhamnosus* CM MSU 529 at the late exponential growth phase. The level of 43 proteins was higher, whereas the level of 14 proteins was lower during aerobic growth compared to static growth (Table 1, Tables S1–S3). The upregulated proteins belonged to the following COG categories: cell cycle control, cell division, chromosome partitioning (D); post-translational modification, protein turnover, and chaperones (O); signal transduction mechanisms (T); defense mechanisms (V); translation, ribosomal structure and biogenesis (J); transcription (K); energy production and conversion (C); amino acid transport and metabolism (E); nucleotide transport and metabolism (F); carbohydrate transport and metabolism (G); coenzyme transport and metabolism (H); lipid transport and metabolism (I); secondary metabolites biosynthesis, transport, and catabolism (Q); general function prediction only (R); function unknown (S). The downregulated proteins were involved in defense mechanisms (V), transcription (K), energy production and conversion (C), amino acid transport and metabolism (E), nucleotide transport and metabolism (F), carbohydrate transport and metabolism (G), and unknown function (S).

The abundance of cell division protein SepF, a cytoplasmic protein forming a division septum in a cytokinesis process by binary fission, increased in aerobic conditions. The production of thioredoxin (TrxA) and its electron acceptor, Tpx-type thiol peroxidase (Tpx), was higher under aerobiosis, while alkyl hydroperoxide reductase C (AhpC), a subunit of an NADH-dependent two-subunit thiol peroxidase complex Ahp, was downregulated upon intensive aeration. The levels of a small stress-induced cytoplasmic ATP-dependent 10-kDa chaperonin (GroES) assisting correct protein folding, a universal stress protein (USP), and a ThiJ/PfpI family protein were increased under aerobiosis. Under aerobic cultivation, the biosynthesis of three proteins involved in translation, ribosomal structure, and biogenesis was upregulated. These are: ribosome-binding factor A (RbfA) assisting in the late steps of the functional core maturation of a free 30S ribosomal subunit, probable tRNA sulfurtransferase (ThiI) catalyzing the ATP-dependent transfer of a sulfur to tRNA to produce 4-thiouridine in tRNAs, and RNA-binding lactamase B domain-containing protein exhibiting exonuclease activity. 

Two transcriptional regulators of the MarR family were involved in the oxidative stress response in *L. rhamnosus* CM MSU 529. The synthesis of organic hydroperoxide resistance transcriptional regulator OhrR was upregulated by aeration, whereas the synthesis of transcriptional regulator homologous to the product of gene LRHMDP2_405 was downregulated by O_2_. 

The abundance of enzymes of energy metabolism, including NADH dehydrogenase, pyruvate dehydrogenase complex E3 component (PdhD), NADH:flavin oxidoreductase (NamA) of Old Yellow Enzyme family (OYE), putative NAD^+^(FAD)-dependent dehydrogenase (Nox-2) reducing O_2_ with H_2_O production, L-lactate oxidase (LctO) oxidizing lactate into pyruvate and H_2_O_2_, increased in aerobic conditions. Overproduction of NADH peroxidase (Npr) and pyruvate oxidase (CidC) was observed under intensive aeration (2.5 and 2.2 log_2_ protein fold change, respectively). By contrast, the amount of NAD^+^-dependent alcohol dehydrogenase oxidizing aromatic alcohols and formate acetyltransferase, or pyruvate formate lyase (PflB), decreased during aerobic growth.

Among proteins involved in the amino acid transport and metabolism, the levels of carbamoyl-phosphate synthase (CarAB) participating in L-arginine and pyrimidine biosynthesis; oligopeptide ABC transporter periplasmic oligopeptide-binding protein OppA, a component of transport system for oligopeptides from the environment; neutral endopeptidase (PepO) hydrolyzing internal polypeptide α–peptide bonds; glycine cleavage system H protein (GcvH) exhibiting the reversible oxidation of glycine to a methylene group; and cysteine synthase (CysK) increased under aerobiosis, while acetolactate synthase (AlsS) converting pyruvate into α-acetolactate and CO_2_ was overproduced (2.9 log_2_ protein fold change). On the contrary, the synthesis of transcriptional regulator, GntR family domain/aspartate aminotransferase (AvtA), involved in production of oxaloacetate from L-aspartate and 2-oxoglutarate; argininosuccinate synthase (ArgG) important for L-arginine biosynthesis; and cell division transporter ATP-binding protein FtsE (GlnQ) with ABC-type glutamine transporter activity was downregulated by aeration.

The levels of enzymes encoded by *pyr* operon, including dihydroorotase (PyrC), aspartate carbamoyltransferase (PyrB), and bifunctional protein PyrR, increased under aerobiosis, while orotate phosphoribosyltransferase (PyrE) was overproduced (2.3 log_2_ protein fold change). The amount of deoxyribose-phosphate aldolase (DeoC), which reversibly degradates 2-deoxy-D-ribose 5-phosphate, a precursor of dCTP and dTTP biosynthesis, increased upon aeration. The synthesis of adenosylcobalamin-dependent ribonucleoside-triphosphate reductase (RtpR) and deoxyadenosine kinase/deoxyguanosine kinase was strongly downregulated upon aeration (−2.0 and −1.1 log_2_ protein fold change, respectively). RtpR converts NDPs/NTPs to dNDPs/dNTPs necessary for DNA synthesis, while deoxyadenosine kinase/deoxyguanosine kinase catalyzes a phosphorilation of 2’-deoxyadenosine/2’-deoxyguanosine into dAMP/dGMP.

The abundance of the following enzymes involved in carbohydrate metabolism was higher under aerobiosis: probable phosphoketolase (Xfp) catalyzing the cleavage of xylulose 5-phosphate into glyceraldehyde 3-phosphate and acetyl-phosphate, pyruvate oxidase (Pox), and glycogen biosynthesis protein GlgD with glucose 1-phosphate adenylyltransferase activity. The level of ABC transporter substrate-binding protein containing sugar binding domain was lower during aerobic growth. The production of citrate lyase β-chain (CitE) significantly decreased in aerobic conditions (−2.9 log_2_ protein fold change).

The synthesis of lipoate-protein ligase (LplA) responsible for protein lipoylation via exogenous pathway, 3-hydroxyisobutyrate dehydrogenase/β-hydroxyacid dehydrogenase reducing succinic semialdehyde/glyoxylate, oxidoreductase of aldo/keto reductase family (DkgA) reducing 2,5-diketo-D-gluconic acid, and α-acetolactate decarboxylase (AlsD) producing acetoin was upregulated upon aeration, while the synthesis of phosphate-binding protein (PstS), part of the ABC transporter complex involved in phosphate import to cell, was downregulated. The predicted pyridoxine 5’-phosphate oxidase V related favin-nucleotide-binding protein (PPO) was overproduced under aerobiosis (2.9 log_2_ protein fold change). PPO is a FMN-binding flavoprotein that catalyzes the oxidation of pyridoxine 5’-phosphate and pyridoxamine 5’-phosphate to pyridoxal 5’-phosphate with the production of H_2_O_2_ in *Escherichia coli* [25].

In spite of the differences mentioned above in proteomic profiles between static and aerated growth conditions, the biomass production and pH values of culture supernatants during static growth were similar to those observed in our previous study during the aerobic growth of strain CM MSU 529 [22]. The biomass was of 2.1 ± 0.1 g dw cells/l and and pH value of culture supernatant was of 3.92 ± 0.03 after 24 h of cultivation in static conditions.

### 3.2. Protein–Protein Interaction Network

**Figure 1:**
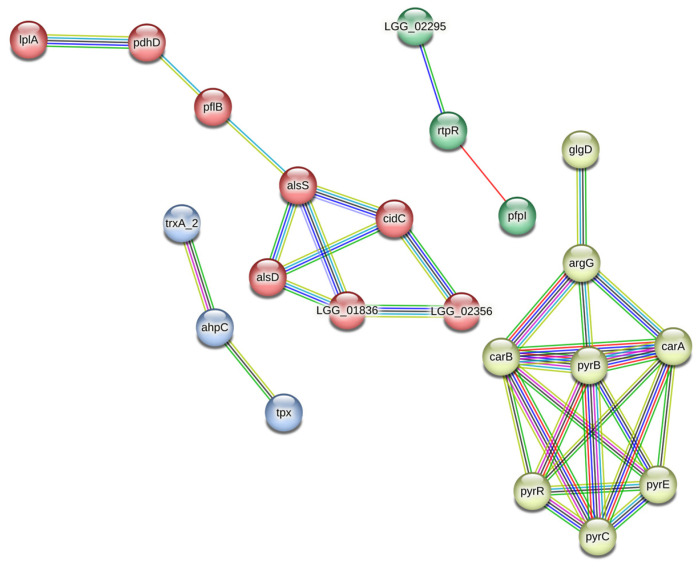
Protein–protein interaction network of differentially abundant proteins in *L. rhamnosus* CM MSU 529. Known interactions: (▬) from curated databases, (▬) experimentally determined. Predicted interactions: (▬) gene neighborhood, (▬) gene fusions, (▬) gene co-occurrence. Others: (▬) textmining, (▬) co-expression, (▬) protein homology. Clusters: (●) 1, (○) 2, (●) 3, (●) 4. LGG_01836 (*pox*), LGG_02356 (*lctO*), and LGG_02295 (LRHMDP2_2713).

Twenty-two of fifty-three differentially abundant proteins were connected in a STRING network indicating functional and physical protein interactions (Figure 1, Tables S5–S8). Cluster analysis revealed 4 independent clusters. The cluster 1 (red) was represented by proteins involved in pyruvate metabolism plus LplA highly associated with lipoate-dependent PdhD. The cluster 2 (white) included highly associated proteins of nucleotide and amino acid metabolism (F/E) plus glycogen biosynthesis protein GlgD (G). DUF4430 domain-containing protein (LGG_02295) of unknown function is encoded by a gene in neighborhood with an *rtpR* gene, whereas RtpR and PfpI are encoded by fused genes in the cluster 3 (green). Components of a thiol-specific antioxidant system are connected into the cluster 4 (blue). 

## 4. Discussion

**Figure 2:**
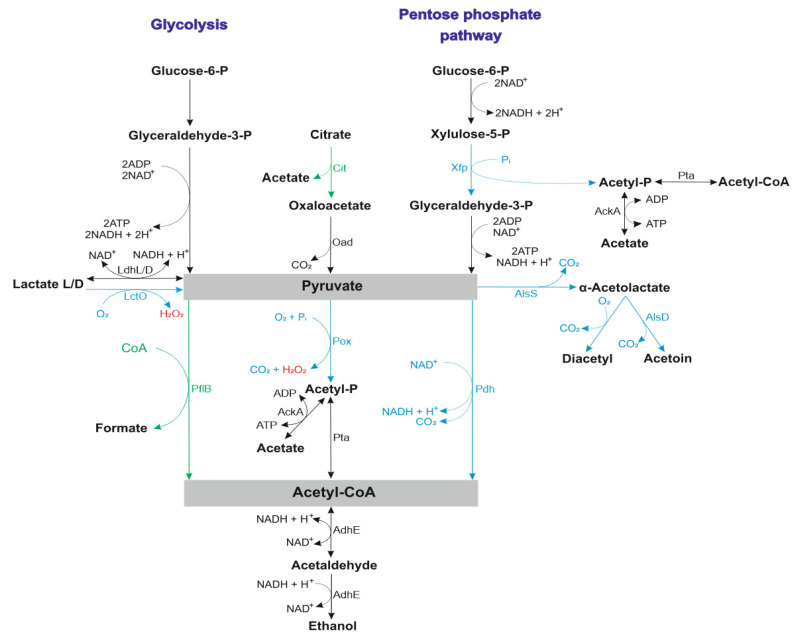
Pyruvate conversion pathways in *L. rhamnosus* CM MSU 529. LdhL/D, lactate dehydrogenase; LctO, lactate oxidase; Cit, citrate lyase; Xfp, phosphoketolase; AckA, acetate kinase; Oad, oxaloacetate decarboxylase; Pox, pyruvate oxidase; PflB, pyruvate formate lyase; Pta, phosphate acetyltransferase; Pdh, pyruvate dehydrogenase complex; AlsS, α-acetolactate synthase; AlsD, α-acetolactate decarboxylase; AdhE, acetaldehyde/alcohol dehydrogenase. Upregulated enzymes are in blue, downregulated enzymes are in green (Table 1, Tables S1–S3), and other identified enzymes are in black (Table S4).

Aeration had a pronounced impact on the proteome of *L. rhamnosus* CM MSU 529. Under intensive aeration, the synthesis of Xfp, a key enzyme of the pentose phosphate pathway, increased (Figure 2). By contrast, the levels of enzymes producing oxaloacetate, CitE and AvtA, decreased in aerobic conditions. These facts imply that there is a shift in pyruvate production routs from the citrate–oxaloacetate pathway towards the pentose phosphate pathway under aerobiosis compared to static growth in *L. rhamnosus* CM MSU 529. Aeration increased the production of Xfp and decreased the production of CitE in *L. casei* N87 as well [20]. Pyruvate is a central intermediate of lactic acid fermentation and can be further converted by lactate dehydrogenase (Ldh) into lactate, by PflB into formate and acetyl-CoA, by Pox into acetyl-phosphate and CO_2_, by Pdh into acetyl-CoA and NADH, and by AlsS into α-acetolactate and CO_2_. Pox, PdhD, AlsS, and AlsD were upregulated during the aerobic growth of strain CM MSU 529, while PflB was downregulated by O_2_. Thus, acetyl-CoA production was mainly due to PflB activity during static growth and due to Pdh, Pox/phosphate acetyltransferase (Pta) or Xfp/Pta activities during aerobic growth. Alternatively in the last two cases, the acetyl-phosphate produced by Pox and Xfp may be further converted to acetate by acetate kinase (AckA) with ATP production. AckA is considered to be important in gaining extra energy under aerobiosis in lactobacilli [13]. Since the abundance of AckA was relatively low in the proteome (score < 50), this reaction does not seem to play a significant role in the energy metabolism of strain CM MSU 529.

The proteome of *L. rhamnosus* CM MSU 529 contains two pyruvate oxidases, CidC and Pox. The synthesis of both enzymes was upregulated by oxygen. The former isoform possesses pyruvate:ubiquinone oxidoreductase activity, whereas the latter isoform oxidizes pyruvate into acetyl-phosphate, CO_2_, and H_2_O_2_. Recently we have shown that strain CM MSU 529 realizes respiratory metabolism in a medium supplemented with hemin and menaquinone [22]. CidC is likely to be a component of the ETC functioning in strain CM MSU 529 under respiratory cultivation. Overexpression of CidC under aerobiosis implies that some components of ETC may be activated by O_2_ in LAB. The expression of the *pox* gene was activated by O_2_ in *Lp. plantarum* Lp80 [26], *Lp. plantarum* WCFS1 [27], *Lt. buchneri* CD034 [17], *L. rhamnosus* N132, *Levilactobacillus spicheri* LP38 [28], *L. casei* N87 [10], and *Levilactobacillus brevis* ATCC 367 [29]. In the last two species, the induction of Pox activity was also observed. Pox5 (PoxB) involved in conversion of pyruvate to acetate was upregulated under aerobiosis versus anaerobiosis in *Lp. plantarum* WCFS1 [19]. In addition to Pox, the synthesis of the H_2_O_2_-producing flavin-dependent oxidases, LctO and predicted PPO, was upregulated by O_2_ in *L. rhamnosus* CM MSU 529. LctO oxidizes lactate into pyruvate, rerouting a final product of lactic acid fermentation back to the carbon and energy metabolism. Oxygen induced transcription of the *lctO* gene in *Lt. buchneri* CD034 [17].

The abundance of AlsS and AlsD increased during aerobic growth. The former converts pyruvate into α-acetolactate and CO_2_, while the latter decarboxylates α-acetolactate into acetoin. Alternatively, α-acetolactate may be converted into diacetyl by nonenzymatical decarboxylation in the presence of oxygen. The proteome of strain CM MSU 529 lacks butanediol dehydrogenase and diacetyl reductase. Als was upregulated by O_2_ in *Leuconostoc gelidum* subsp. *gelidum* TMW2.1618 and in *Ln*. *gelidum* subsp. *gasicomitatum* TMW2.1619 grown in a heme-supplemented medium [21].

*L. rhamnosus* CM MSU 529 contains two thiol-specific peroxidases, TrxA-dependent Tpx-type and NADH-dependent AhpC, which synthesis was up- and downregulated by O_2_, respectively. The production level of TrxA, a small disulphide-containing protein, was higher under aerobiosis. Since reduced TrxA possesses a Tpx-disulfide reductase activity, it provides the Tpx functioning [13]. Upregulated by O_2_ NADH dehydrogenase with FAD/NAD(P)^+^-binding domain (K8Q7J1) might play a role of TrxA reductase supporting an intracellular disulphide/thiol balance in strain CM MSU 529. In addition to Tpx, production of Npr significantly increased under aerobiosis. Both peroxidases play a key role in the effective elimination of H_2_O_2_ formed by oxidases (LctO, Pox, and PPO), which synthesis was induced during aerobic growth. Genes encoding TrxA, Tpx, and Npr are widely spread among lactobacilli [13]. *L*. *rhamnosus* lacks genes encoding catalase and superoxide dismutase. Thus, Tpx and Npr participate in detoxification of H_2_O_2_ and organic hydroxyperoxides predominantly in aerobic conditions, whereas AhpC carries out this function during the static growth of strain CM MSU 529. The *ahpC* gene expression was upregulated by cold in *Lc. lactis* MCC866 cocultivated with bifidobacteria after refrigerated storage in fermented milk [30]. The synthesis of AhpC was cold-inducible in *Leuconostoc mesenteroides* NH04 [31]. NADH peroxidase Npr2 was upregulated under aerobiosis versus anaerobiosis in *Lp. plantarum* WCFS1 [19].

The CysK level increased upon intensive aeration in strain CM MSU 529. This observation is in a good agreement with the upregulation of TrxA and Tpx biosynthesis, since these proteins harbor Cys residues in their active centers. In addition, Cys is a strong antioxidant itself [32]. Overexpression of the predicted PPO under aerobiosis is likely to support the biosynthesis of pyridoxal 5’-phosphate-dependent CysK in strain CM MSU 529. The amount of CysK increased in a controlled batch culture of *L. casei* N87 under aerobiosis versus anaerobiosis [20].

DkgA catalyzes the NADPH-dependent reduction of 2,5-diketo-D-gluconic acid to 2-keto-L-gulonic acid, a key intermediate in the synthesis of L-ascorbic acid. Thus, the higher amount of DkgA under aeration may enhance the antioxidant properties of strain CM MSU 529.

The biosynthesis of proteins involved in the general stress response (GroES, USP, PfpI, and YqiG) was upregulated by O_2_ in *L. rhamnosus* CM MSU 529. The putative heat shock protein GroES was among the proteins whose synthesis was induced in response to acid conditions in *Lactobacillus acidophilus* CRL 639 [33] and *Lactobacillus bulgaricus* [34]. The role and the mechanism of action of the USPs in Gram-positive bacteria are still obscure. The *usp1* gene was overexpressed during the the phenolic acid stress response in *Lp. plantarum* [35]. The capacity of Usp1 to inactivate the repressor of the phenolic acid stress response PadR implies that it could be a mediator in the acid stress response mechanism. The ThiJ/PfpI family proteins are suggested to be involved in cellular protection against various environmental stresses [36]. The members of the ThiJ/PfpI family are diverse in structure and function and include heat-shock protein 31 (Hsp31), a chaperone and a peptidase of *E. coli* [37]; PH1704, a thermophilic protease/peptidase of archaean *Pyrococcus horikoshii* OT3 [38]; and YhbO involved in the response to hyperosmotic and acid stresses in *E. coli* [39]. Hsp31 paralog with glyoxalase activity was demonstrated to combat oxidative stress in *Saccharomyces cerevisiae* [40]. FMN-dependent OYEs reduce a wide range of activated C=C bonds in α,β-unsaturated carbonyl compounds to their saturated counterparts [41] and were reported to participate in stress response. Thus, the expression of *ofrA* gene encoding OYE of *Staphylococcus aureus* was induced by hypochlorite, oxidative, and electrophilic stresses [42]. YqiG appears to be important in maintaining an intracellular redox balance reducing the electrophilic carbonyl compounds imposed by intensive aeration in strain CM MSU 529. Upregulated upon aeration NADP^+^-dependent 3-hydroxyisobutyrate dehydrogenase related β-hydroxyacid dehydrogenase should be also considered as the general stress response protein. The function of 3-hydroxyisobutyrate dehydrogenases/β-hydroxyacid dehydrogenases reducing succinic semialdehyde and glyoxylate to *γ*-hydroxybutyrate and glycolate, respectively, has been proposed to detoxify both aldehydes during stress response in *Arabidopsis* [43]. Phylogenetically related glyoxylate reductases/succinic semialdehyde reductases were found in bacteria as well. The purified 3-hydroxyisobutyrate dehydrogenase-type proteins from *Geobacter* spp. [44] and *Gluconobacter oxydans* 621H [45] revealed succinic semialdehyde reductase activities. In addition, the NAD(P)H-dependent glyoxylate reductase with high substrate specificity for glyoxylate was recently characterized from *Acetobacter aceti* JCM20276 [46].

The increased production of RbfA and RNA-binding VicX may be important for the maturation and stability of the free 30S and 70S ribosomal subunit in strain CM MSU 529 under aerobiosis, respectively. ThiI transfers a sulfur to tRNA and to the sulfur carrier protein ThiS, forming ThiS-thiocarboxylate, which is a stage in the synthesis of thiazole in the thiamine diphosphate (TPP) biosynthetic process. Thus, the higher level of the ThiI supports the upregulated synthesis of TPP-dependent enzymes (Pox, AlsS) in *L. rhamnosus* CM MSU 529 during aerobic growth. Interestingly, the Fe-S biosynthesis domain-containing protein (YkuJ) with unknown function may be involved in the [2Fe-2S] cluster synthesis of ferredoxin needed for sulfurtransferase activity of ThiI.

Two transcriptional regulators of the MarR family changed in amount in *L. rhamnosus* CM MSU 529 upon aeration. OhrR was involved in the oxidative stress response, while the transcriptional regulator homologous to the gene LRHMDP2_405 product was downregulated upon intensive aeration. The MarR family transcriptional regulators are known to be repressors of genes activating the oxidative stress regulons. OhrR is a transcriptional repressor of the organic hydroperoxide resistance protein (OhrA). The gene *ohrR* deficient mutant revealed a strong resistance to H_2_O_2_ in *L. casei* IGM394 [47]. OhrA was not detected in this study.

The level of PdhD increased upon aeration in strain CM MSU 529. The amount of PdhD also increased in the proteome of *L. casei* N87 in a controlled batch culture under aerobiosis compared to anaerobiosis [20]. The expression of *pdh* genes, as well as Pdh activity, was upregulated by oxygen in *Lv. brevis* ATCC 367 at the late exponential growth phase [29]. 

GcvH is a component of the glycine cleavage system catalyzing the reversible oxidative cleavage of glycine to a methylene group, ammonia, and CO_2_. Thus, the increased level of GcvH may maintain pH homeostasis in *L. rhamnosus* CM MSU 529 under aerobiosis. Another mechanism that prevents the intracellular acidification is the upregulation upon aeration of enzymes of the pyruvate–diacetyl/acetoin pathway, AlsS and AlsD. This pathway contributed to pH homeostasis in acid stress conditions in *Lc. lactis* [48].

LplA responsible for ATP-dependent protein lipoylation via the exogenous pathway was upregulated upon aeration in strain CM MSU 529. This fact is in good agreement with upregulation of the lipoate-requiring proteins, GcvH and PdhD.

The abundance of CarAB increased under aerobic compared to static conditions, whereas the production of ArgG decreased under intensive aeration in strain CM MSU 529. The arginine and pyrimidine biosynthesis pathways have a common precursor, carbamoyl-phosphate. This implies that there is a shift from L-arginine synthesis towards pyrimidine nucleotides synthesis in lactic acid bacterium under aerobic conditions. The higher levels of PyrE, PyrC, PyrB, and PyrR involved in pyrimidine biosynthesis in aerobic conditions compared to static growth support this conclusion. The carbamoyl-phosphate and uridine 5′-monophosphate (UMP) synthesis depends on concentration of the dissolved form of CO_2_ (HCO_3_^−^) in a growth medium. A high requirement of CO_2_ for arginine and pyrimidine biosynthesis was found in 74 of 207 tested strains of lactobacilli [49]. Therefore, the upregulation of CO_2_-producing enzymes (Pox, AlsS, AlsD, and GcvH) favors the pyrimidine nucleotides biosynthesis in strain CM MSU 529 during aerobic growth. The upregulation of CarAB and PyrECBR in *L. rhamnosus* CM MSU 529 by O_2_ is rather unusual. The expression of the *carB* and *pyrC* genes was downregulated by aeration in *Lc. lactis* [15]. The amount of CarB decreased in a controlled batch culture of *L. casei* N87 under aerobiosis compared to anaerobiosis [20]. The abundance of PyrC was lower in oxic versus anoxic conditions in *Carnobacterium divergens* TMW2.1577 grown in a meat simulation medium [21]. The upregulation PyrECBR was not followed by an increase in biomass production of *L. rhamnosus* CM MSU 529 under aerobiosis. Instead, an increase in the level of GlgD promoted the activation of glycogen biosynthetic process by aeration in strain CM MSU 529.

DeoC reversibly degradates 2-deoxy-D-ribose 5-phosphate to D-glyceraldehyde 3-phosphate and acetaldehyde providing both the synthesis and catabolism of nucleosides. In the latter case, D-glyceraldehyde 3-phosphate enters the central carbon metabolism pathways as a carbon and energy source (Figure 2). *E. coli* mutants lacking DeoC were not able to catabolize the deoxyribose moiety of deoxyribonucleosides, while there were no significant alterations in the cellular pools of NTPs and dNTPs [50], suggesting a catabolic function of DeoC. The activities of DeoC isolated from *Lp. plantarum* and a rat liver were highly dependent on the presence of polycarboxylic acids [51,52]. In this connection, the downregulation of CitE upon aeration in strain CM MSU 529 may favor the activation of DeoC by citrate during aerobic growth. Interestingly, the amount of DeoC was lower under aerobiosis in comparison with anaerobiosis in *C. divergens* TMW2.1577 [21].

Ribonucleosidereductase reduces nucleosides (U, A, G, and C) to the corresponding deoxynucleosides [53]. The adenosylcobalamin-dependent RtpR of strain CM MSU 529 catalyzes this reaction at the level of NDP and NTP using TrxA as an electron donor. The underexpression of RtpR in aerobiosis may help to maintain the high levels of NDPs/NTPs needed for RNA biosynthesis to support the production of the upregulated by aeration enzymes. The downregulation of deoxyadenosine kinase/deoxyguanosine kinase results in decreased concentrations of dAMP/dGMP, the precursors of dATP/dGTP, during the aerobic growth of strain CM MSU 529. This fact is in good agreement with the low level of PstS under aeration. 

OppA, a surface located protein in lactobacilli, is a component of the transport system for oligopeptides from the environment for nutrition. The amounts of OppA and PepO increased, while the abundance of GlnQ decreased under aerobiosis, suggesting rerouting of nutrition from amino acids to oligopeptides upon aeration in strain CM MSU 529. The enhanced import of oligopeptides appears to support the biosynthesis of upregulated proteins in lactic acid bacterium under aerobic conditions. The expression of the *oppa3* gene was upregulated under aerobic versus anaerobic conditions in *Lt. buchneri* CD034 [17]. The *pepO1* expression was stimulated by aeration compared to static conditions in *Lc. lactis* MG1363 [18]. The *glnQ* gene expression was downregulated in *Lc. lactis* in aerobic conditions compared to static conditions [14].

The upregulation of NADH dehydrogenase (gene LRHMDP2_2182), YqiG, Nox-2, Npr diminishes an intracellular level of NADH providing cell redox homeostasis (NAD^+^/NADH) in strain CM MSU 529 under aerobiosis.

Proteins of *L. rhamnosus* CM MSU 529 were identified by comparative analysis with the *L. rhamnosus* LRHMDP2 proteome. Sequencing of the entire genome of strain CM MSU 529 may lead to a deeper knowledge of its protein profile.

## 5. Conclusions

Proteomic analysis is a powerful tool to elucidate the molecular mechanisms of cellular adaptation to various environmental conditions. The significant difference in the proteomic profile of aerobically grown cells compared to statically grown cells of *L. rhamnosus* CM MSU 529 was observed. Aerobic cultivation caused the induction of enzymes responsible for oxygen consumption, xylulose 5-phosphate and pyruvate conversion, ROS defense, general stress response, antioxidant production, pyrimidine biosynthesis, oligopeptide transport and metabolism, ribosome stabilization, intracellular pH and redox homeostasis. In spite of the fact that intensive aeration impaired the citrate–pyruvate pathway, arginine and DNA biosynthesis, it had no effect on the biomass production. The metabolism of *L. rhamnosus* CM MSU 529 is therefore precisely tuned to the standing concentration of O_2_ in such a way that the strain is able to maintain energy conservation enough for achieving optimal biomass production under intensive aeration. Further elucidation of the influence of aerobic cultivation on probiotic properties, cell survival to a long-term storage and environmental stresses is important in light of the prospect of industrial application of a strain CM MSU 529 aerobic phenotype. The presented results provide new knowledge on the molecular mechanisms employed by LAB in adaptation to aerobic growth conditions.

## Figures and Tables

**Table 1:** Differentially expressed proteins of *L. rhamnosus* CM MSU 529 in aerobic conditions compared to static conditions.

Identified Proteins	Protein AC	Gene	MW, kDa	log_2_ Fold Change A/S ^1^
CELLULAR PROCESSES AND SIGNALING				
[D] Cell cycle control, cell division				
Cell division protein SepF	K8QLA2	*sepF*	16.8	0.6
[O] Post-translational modification, protein turnover, and chaperones				
Thioredoxin	K8Q3Z2	*trxA, trxA_2*	11.5	1.2
Thiol peroxidase Tpx-type	K8QQU7	*tpx*	18.5	1.6
10 kDa chaperonin	K8QSC4	*groES, groS*	10.0	0.9
[T] Signal transduction mechanisms				
Universal stress protein	K8QLD2	LRHMDP2_1718	18.2	0.7
[V] Defense mechanisms				
ThiJ/PfpI family protein	K8QE31	*yfkM, pfpI*	18.4	0.7
Alkyl hydroperoxide reductase C	K8QI55	*ahpC*	20.5	−0.9
INFORMATION STORAGE AND PROCESSING				
[J] Translation, ribosomal structure and biogenesis				
Ribosome-binding factor A	K8QHI9	*rbfA*	16.7	0.7
Probable tRNA sulfurtransferase	K8QLD7	*thiI*	45.2	0.6
Lactamase_B domain-containing protein	K8QEJ1	*vicX*	46.3	0.9
[K] Transcription				
Organic hydroperoxide resistance transcriptional regulator	K8QM79	*ohrR*	16.1	0.8
Transcriptional regulator, MarR family	K8QLH4	LRHMDP2_405	17.4	−0.6
METABOLISM				
[C] Energy production and conversion				
NADH dehydrogenase	K8Q7J1	LRHMDP2_2182	42.7	1.0
Dihydrolipoyl dehydrogenase (E3)	K8QFD2	*pdhD, lpdA*	49.1	1.2
NADH:flavin oxidoreductase Old Yellow Enzyme family	K8Q900	*namA, yqiG*	41.8	1.3
Putative NAD^+^(FAD)-dependent dehydrogenase	K8QIY4	*nox_2*	49.1	1.7
L-lactate oxidase	K8Q6V5	*lctO*	39.3	1.2
NADH peroxidase Npx	K8QQ66	*npx, npr*	49.3	**2.5**
Pyruvate oxidase [C/H/R]	K8QGX3	*ydaP, cidC*	62.7	**2.2**
Alcohol dehydrogenase	K8QNN2	*xylB_1, xylB_3*	39.8	−1.6
Formate acetyltransferase	K8QD01	*pflB*	85.2	−1.4
[E] Amino acid transport and metabolism				
Carbamoyl-phosphate synthase small chain [E/F]	K8Q6G7	*carA*	39.4	0.9
Carbamoyl-phosphate synthase large chain [E/F]	K8QCB0	*carB*	116.2	1.5
Acetolactate synthase catabolic [E/H]	K8QB15	*alsS*	60.5	**2.9**
Oligopeptide ABC transporter periplasmic oligopeptide-binding protein OppA	K8QLE7	*oppA, oppA_2*	60.4	1.0
Neutral endopeptidase	K8Q9I4	*pepO*	71.7	0.6
Glycine cleavage system H protein	K8QLC1	*gcvH*	10.8	0.6
Cysteine synthase	K8QQD2	*cysK*	32.6	0.7
Transcriptional regulator, GntR family domain/Aspartate aminotransferase [E/K]	K8Q1F8	*avtA*	44.2	−0.9
Argininosuccinate synthase	K8QAT7	*argG*	44.7	−0.7
Cell division transporter ATP-binding protein FtsE	K8QCV1	*glnQ*	27.3	−0.7
[F] Nucleotide transport and metabolism				
Orotate phosphoribosyltransferase	K8Q3P7	*pyrE*	22.4	**2.3**
Dihydroorotase	K8Q3Q2	*pyrC*	45.1	1.8
Aspartate carbamoyltransferase	K8QFX4	*pyrB*	35.1	1.4
Bifunctional protein PyrR	K8QCB4	*pyrR*	19.2	1.6
Deoxyribose-phosphate aldolase	K8QGF6	*deoC*	23.0	1.1
Adenosylcobalamin-dependent ribonucleoside-triphosphate reductase	K8Q4T0	*rtpR, nrdJ*	82.5	**−2.0**
Deoxyadenosine kinase/Deoxyguanosine kinase	K8QBP7	LRHMDP2_1731	27.2	−1.1
[G] Carbohydrate transport and metabolism				
Probable phosphoketolase	K8Q7D7	*xfp, xpkA*	89.8	1.2
Glycogen biosynthesis protein GlgD, glucose-1-phosphate adenylyltransferase family	K8QJ10	*glgD*	43.1	0.9
Pyruvate oxidase [G/H/R]	K8Q595	*spxB, pox*	64.0	1.0
ABC transporter substrate-binding protein	K8QJU7	LRHMDP2_357	34.0	−1.1
Citrate lyase beta chain	K8Q549	*citE*	31.4	**−2.9**
[H] Coenzyme transport and metabolism				
Lipoate-protein ligase	K8Q5G3	*lplJ, lplA*	38.4	0.9
[I] Lipid transport and metabolism				
3-hydroxyisobutyrate dehydrogenase related beta-hydroxyacid dehydrogenase	K8QD39	LRHMDP2_2289	30.1	1.3
[P] Inorganic ion transport and metabolism				
Phosphate-binding protein	K8QH56	*pstS*	32.0	−0.8
[Q] Secondary metabolites biosynthesis, transport, and catabolism				
Oxidoreductase of aldo/keto reductase family, subgroup 1	K8Q5E9	*dkgA, yqhE*	31.6	0.7
Alpha-acetolactate decarboxylase	K8QD20	*budA, alsD*	25.7	1.2
POORLY CHARACTERIZED				
[R] General function prediction only				
Pyridoxine 5’-phosphate oxidase V related favin-nucleotide-binding protein	K8Q7A0	LRHMDP2_2560	14.7	**2.9**
[S] Function unknown				
UPF0297 protein	K8QCI9	*alaRS*	10.1	1.1
UPF0291 protein	K8QHM8	*ynzC*	9.7	0.7
DUF124 domain-containing protein	K8QS65	*yfhL*	25.9	0.9
Multi-ubiq domain-containing protein	K8Q6T6	LRHMDP2_1960	20.1	1.7
YfIT domain-containing protein	K8QDE0	LRHMDP2_784	14.3	1.2
Fe-S_biosyn domain-containing protein	K8Q441	*ykuJ*	10.5	0.9
Putative pheromone lipoprotein (FMN-binding domain protein)	K8Q706	*cad*	32.6	−0.9
Additional lipoprotein component of putative cobalamin ECF transporter	K8Q211	LRHMDP2_2713	14.3	−1.8

^1^ log_2_ Fold Change A/S, log_2_ protein fold change in aerobic versus static condition; log_2_ fold change values of significantly differentially abundant proteins are in bold. Upregulated proteins are in yellow; downregulated proteins are in blue.

## Data Availability

The mass spectrometry proteomics data were deposited to the ProteomeXchange Consortium via the PRIDE (http://www.ebi.ac.uk/pride, accessed on 15 December 2022) partner repository with the dataset identifier PXD038847.

## Supplementary Materials

The following supporting information can be downloaded at: https://www.mdpi.com/article/10.3390/microorganisms11020313/s1, Table S1: Differentially expressed proteins of *L. rhamnosus* CM MSU 529 in aerobic conditions versus static conditions; Table S2: MaxQuant analysis of differentially expressed proteins of *L. rhamnosus* CM MSU 529 grown under static and aerobic conditions; Table S3: Proteins fold change and statistical analyses on differentially expressed proteins of *L. rhamnosus* CM MSU 529 in aerobic versus static conditions; Table S4: Enzymes of pyruvate metabolism of *L. rhamnosus* CM MSU 529; Table S5: STRING annotations of differentially abundant proteins of *L. rhamnosus* CM MSU 529; Table S6: STRING functional annotations of differentially abundant proteins of *L. rhamnosus* CM MSU 529; Table S7: Node degrees in a STRING network; Table S8: Protein–protein interactions of differentially abundant proteins in *L. rhamnosus* CM MSU 529.
